# A nomogram based on the expression level of angiopoietin-like 4 to predict the severity of community-acquired pneumonia

**DOI:** 10.1186/s12879-023-08648-4

**Published:** 2023-10-11

**Authors:** Siqin Chen, Jia Jiang, Minhong Su, Ping Chen, Xiang Liu, Wei Lei, Shaofeng Zhang, Qiang Wu, Fu Rong, Xi Li, Xiaobin Zheng, Qiang Xiao

**Affiliations:** 1 Pulmonary and Critical Care Medicine, Shunde Hospital, Southern Medical University, No.1, Jiazi Road, Lunjiao Street, Shunde District, Foshan, 528300 China; 2 Pulmonary and Critical Care Medicine, Zhujiang Hospital, Southern Medical University, Guangzhou, China; 3 GMU-GIBH Joint School of Life Sciences, The Guangdong-Hong Kong-Macau Joint Laboratory for Cell Fate Regulation and Diseases, Guangzhou Medical University, Guangzhou, China; 4 Departments of Hematology, Shunde Hospital, Southern Medical University, Foshan, China; 5 Department of Cardiology, National Clinical Research Center for Infectious Disease, State Key Discipline of Infectious Disease, Shenzhen Third People’s Hospital, Second Hospital Affiliated to Southern University of Science and Technology, Shenzhen, China; 6 Pulmonary and Critical Care Medicine, The Fifth Affiliated Hospital of Sun Yat-Sen University, 52 East Meihua Rd., Zhuhai, 519000 China

**Keywords:** Angiopoietin-like 4 (ANGPTL4), Severe community-acquired pneumonia, Biomarker, Precision medicine, Prediction model

## Abstract

**Background:**

The morbidity and mortality of community-acquired pneumonia (CAP) remain high among infectious diseases. It was reported that angiopoietin-like 4 (ANGPTL4) could be a diagnostic biomarker and a therapeutic target for pneumonia. This study aimed to develop a more objective, specific, accurate, and individualized scoring system to predict the severity of CAP.

**Methods:**

Totally, 31 non-severe community-acquired pneumonia (nsCAP) patients and 14 severe community-acquired pneumonia (sCAP) patients were enrolled in this study. The CURB-65 and pneumonia severity index (PSI) scores were calculated from the clinical data. Serum ANGPTL4 level was measured by enzyme-linked immunosorbent assay (ELISA). After screening factors by univariate analysis and receiver operating characteristic (ROC) curve analysis, multivariate logistic regression analysis of ANGPTL4 expression level and other risk factors was performed, and a nomogram was developed to predict the severity of CAP. This nomogram was further internally validated by bootstrap resampling with 1000 replications through the area under the ROC curve (AUC), the calibration curve, and the decision curve analysis (DCA). Finally, the prediction performance of the new nomogram model, CURB-65 score, and PSI score was compared by AUC, net reclassification index (NRI), and integrated discrimination improvement (IDI).

**Results:**

A nomogram for predicting the severity of CAP was developed using three factors (C-reactive protein (CRP), procalcitonin (PCT), and ANGPTL4). According to the internal validation, the nomogram showed a great discrimination capability with an AUC of 0.910. The Hosmer–Lemeshow test and the approximately fitting calibration curve suggested a satisfactory accuracy of prediction. The results of DCA exhibited a great net benefit. The AUC values of CURB-65 score, PSI score, and the new prediction model were 0.857, 0.912, and 0.940, respectively. NRI comparing the new model with CURB-65 score was found to be statistically significant (NRI = 0.834, *P* < 0.05).

**Conclusion:**

A robust model for predicting the severity of CAP was developed based on the serum ANGPTL4 level. This may provide new insights into accurate assessment of the severity of CAP and its targeted therapy, particularly in the early-stage of the disease.

## Introduction

Community-acquired pneumonia (CAP) is a pulmonary parenchymal inflammation due to infections acquired outside the hospital. As one of the leading causes of morbidity and mortality worldwide, it has been the major burden of health resources [1, 2]. A cohort study in the USA reported an incidence of CAP in the intensive care unit (ICU) of 145 cases per 100,000 adults per year [3]. The untimely ICU care or the inappropriate administration of antibiotic drugs due to incorrect assessment of the severity of CAP is associated with poor prognosis. When pulmonary inflammation is too severe and spreads too fast, it will evolve into the stage called severe CAP (sCAP), which usually accompanied with respiratory failure, circulatory failure or multiple organ failures. However, there is a lack of a standard definition for sCAP [4]. Generally, patients who need mechanical ventilation or vasopressor support of shock are considered to have severe CAP (sCAP) according to the consensus guidelines from the Infectious Disease Society of America (IDSA) and American Thoracic Society (ATS) [5–7]. In addition to the above-mentioned standards, the pneumonia severity index (PSI) and CURB-65 score are widely recommended for assessment of the severity of CAP. PSI is one of the most all-round scoring systems for grading the severity of CAP [8], while it is extremely complex to be friendly used based on 20 variables. The CURB-65 score is simple for use, including only five scoring items: consciousness disorders, uremia, respiratory rate, blood pressure, and age [9]. However, it may have low specificity in certain populations, such as the elderly due to the unapparent symptoms in the early-stage of the disease and the non-specific laboratory indicators [10]. Some studies included biomarkers, such as C-reactive protein (CRP) and procalcitonin (PCT) into the scoring system to improve the prediction performance, whereas their results were unsatisfactory [11–13]. Therefore, developing a more specific and accurate scoring system for assessment of the severity of CAP is of great significance, thereby leading to effectively improve treatment and prognosis of CAP.

Biomarkers are recognized to be in a prominent position in disease diagnosis and assessment, and the combination of biomarkers with scoring systems seems to be more in line with the era of precision medicine. Host inflammatory response is the most important factor in the process of respiratory infection diseases, which may finally lead to lung damage [14]. The onset and severity of pneumonia are primarily determined by the balance between pathogenic factors (virulence, bacterial abundance) and host factors [15]. In recent years, great attention has been paid to the host response to infection, which was regarded as a turning point in disease diagnosis and treatment, especially in terms of inflammation-related molecular biology [16–19]. Up to now, many biomarkers about pneumonia have been developed, among which CRP and PCT are most commonly used in clinical practice. But they are not ideal biomarkers due to the lack of specificity for lung inflammation and the susceptibility to confused measurement factors. Better than CRP and PCT, there are many novel host-response biomarkers, such as soluble triggering receptor expressed on myeloid cells-1 (sTREM-1), pro-adrenomedullin (proADM) and Presepsin, which have been proved more accurate in evaluating sCAP [11, 20–25]. However, it is not yet clear whether the changes of the above markers are specific to pneumonia. Moreover, there are no biomarkers that could correctly diagnose CAP alone, and the search for the ideal pneumonia biomarker is still ongoing.

Recently, the role of angiopoietin-like 4 (ANGPTL4) expression level in lung diseases has noticeably attracted scholars’ attention. It was found that ANGPTL4 expression level was upregulated in pneumonia, and it was involved in angiogenesis and regulation of vascular permeability, inflammatory response, lung tissue leakiness and injury, lipid and carbohydrate metabolism, wound healing, tumorigenesis, etc. [14, 26]. It was reported that ANGPTL4 expression level was significantly elevated in mouse models and patients with infectious pneumonia [27]. Moreover, the monomeric C-terminal portion (cANGPTL4), released from proteolytic procession of ANGPTL4, was specifically upregulated in the injured lung tissue, and the cANGPTL4 expression level was highly consistent with the degree of lung tissue damage [27]. Compared to other biomarkers, its specific expression in injured lung tissue appears to be more valuable in evaluating the severity of CAP. According to its key role in lung infection and damage, ANGPTL4 may serve as a highly potential marker for diagnosis, severity assessment, and therapy of CAP patients [14, 27].

To date, no study has used ANGPTL4 expression level to quantify the severity of CAP. Nomograms have been newly used to estimate the probability of an event, such as diagnosis, severity, and death, which is presented as a user-friendly graph [28]. It is tailored to individuals and is in line with the trend of precision medicine. In order to establish a more objective, specific, accurate, and individualized scoring system, the present study attempted to combine serum ANGPTL4 expression level with clinical data, such as symptoms, signs, laboratory indicators, and radiologic examinations to develop a nomogram for predicting the severity of CAP, which may assist to provide timely and effective treatment for the early-stage CAP and may promote the development of precision medicine.

## Materials and methods

### Data sources

Totally, 45 patients with CAP who were admitted to the Shunde Hospital of Southern Medical University (Foshan, China) were enrolled in this study. The diagnostic criteria for CAP were summarized as follows: onset in the community; clinical manifestations of pneumonia (recent cough, expectoration, or exacerbated symptoms of the previous respiratory illnesses, with or without chest pain, dyspnea, or hemoptysis; fever; evidences of pulmonary consolidation and/or moist rales in auscultation; peripheral white blood cell (WBC) count > 10*10^9^/L or < 4*10^9^/L); and a new infiltrate of lungs on the chest radiograph. These patients were divided into two groups according to the consensus guidelines from the IDSA/ATS, in which patients who needed mechanical ventilation or vasopressor support of shock were considered to have sCAP [5, 7]. Patients with nosocomial infection, non-infectious interstitial lung disease, malignancy, active pulmonary tuberculosis, and severe immunosuppression were excluded. The clinical data were collected from the electronic medical record system of our hospital, including baseline characteristics of patients, symptoms, signs, laboratory indicators, and radiological data. According to the corresponding scoring criteria, the CURB-65 and PSI scores of each participant were calculated. The present study was approved by the Institutional Human Research and Ethics Committee of the Shunde Hospital of the Southern Medical University. All participants were informed about the purpose of the study and informed consent was obtained from all participants before enrollment.

### Blood sampling and determination of ANGPTL4 expression level

Blood samples were obtained from all participants on the first day of hospitalization using vacutainer tubes with a clot activator, which were coagulated at 25 ℃ for 30 min, and were then centrifuged at 3000 × g for 5 min. The serum samples were collected from the supernatant and preserved at -80 ℃ until analysis. ANGPTL4 expression level was measured using an enzyme-linked immunosorbent assay (ELISA) kit (Cat. No. ab99974; Abcam, Shanghai, China).

### Statistical analysis

SPSS 26.0 (IBM Corp., Armonk, NY, USA) and R (Ver. 3.6.2) software were used to perform statistical analysis. The Shapiro–Wilk test was applied for assessment of the distribution of the measurement data, and Levene’s test was utilized for indicating the equality of variances. The normally distributed data were expressed as mean ± standard deviation, and the independent-sample t-test was used for making comparison between the two groups. The abnormally distributed measurement data were presented as median (interquartile range), and the non-parametric Mann–Whitney U test was used for making comparison between the two groups. The categorical variables were described as count and percentage, and the Chi-square test was used for making comparison. Every factor that might affect the severity of pneumonia, along with CURB-65 and PSI scores, was firstly analyzed by t-test, Mann–Whitney U test, and Chi-square test. The risk factors that had significant difference (*P* < 0.05) were further screened by the receiver operating characteristic (ROC) curve analysis. The statistically significant variables with an area under the ROC curve (AUC) > 0.8 were selected to fit a multivariate logistic regression model. Then, the nomogram was developed with independent risk factors according to the results of multivariate logistic regression analysis. The prediction performance of the nomogram was internally validated by bootstrap resampling with 1000 replications. The discrimination of sCAP patients from CAP patients was carried out by the ROC curve analysis. The calibration and the Hosmer–Lemeshow test, were carried out to validate the accuracy of the multiple logistic regression model. Decision curve analysis (DCA) was employed to assess the clinical utility of the prediction nomogram. Then, the AUC values of CURB-65 score, PSI score, and the new nomogram including ANGPTL4 were compared by DeLong’s test to assess the discrimination ability of different scoring systems. The net reclassification index (NRI) and integrated discrimination improvement (IDI) were calculated to compare the prediction accuracy of different scoring systems. *P* < 0.05 was considered statistically significant.

## Results

### Univariate analysis of demographic and clinical characteristics

A total of 45 patients were included in the present study (31 patients in the non-sCAP (nsCAP) group and 14 patients in the sCAP group). As shown in Table 1, patients in the sCAP group had higher pulse and lower systolic blood pressure and diastolic blood pressure than those with mild pneumonia (*P* < 0.05). Among sCAP patients, the number of cases with consciousness disorders and pleural effusion was higher (*P* < 0.001). Patients with sCAP were more likely to have lower hematocrit and platelet levels and higher levels of neutrophils, CRP, PCT, Na + , glucose, blood urea nitrogen, and ANGPTL4 (*P* < 0.05). The distribution of gender and age between the two groups was not significantly different. The comorbid conditions and other signs besides pulse and blood pressure were similar between the two groups. There was no significant difference in the WBC count, neutrophil count, and levels of pH and K + between the two groups.

**Table 1:** Characteristics of nsCAP and sCAP participants

Characteristic	nsCAP	sCAP	Univariable analysis	
	(*n* = 31)	(*n* = 14)	*χ2*/t/U	*P*-value
Gender			0.551	0.458
Female	10(32.3%)	3(21.4%)		
Male	21(67.7%)	17(78.6%)		
Age, years	60.87 ± 14.79	61.36 ± 17.84	-0.096	0.924
Comorbid conditions				
Diabetes mellitus	4(12.9%)	4(28.6%)	0.725	0.394
Cancer	1(3.2%)	3(21.4%)	2.018	0.155
Chronic liver	3(9.7%)	2(14.3%)	0	1
Congestive heart failure	2(6.5%)	1(7.1%)	0	1
Cerebrovascular	2(6.5%)	3(21.4%)	0.936	0.333
Signs				
Temperature,℃	36.8 (36.7,37.3)	37.15 (36.7,37.7)	-0.775	0.438
Pulse, bpm	87 (78,102)	101.5 (94.25,109.5)	-1.987	0.047*
Respiratory rate, Breaths per minute	20 (20,22)	18 (15.75,25)	-1.751	0.08*
SBP, mmHg	124 (113,137)	112 (104,124.25)	2.281	0.023*
DBP, mmHg	78.26 ± 12.68	59.29 ± 8.87	5.052	< 0.001*
Consciousness disorders	2(6.5%)	12(85.7%)	24.694	< 0.001*
Pleural effusion	7(22.6%)	11(78.6%)	12.598	< 0.001*
Laboratory examination				
WBC counts, x10^9/L	9.56 (7.16,18.00)	12.64 (8.80,20.79)	-1.128	0.259
Neutrophil counts, x10^9/L	7.52 (5.22,14.50)	10.57 (7.29,18.39)	-1.582	0.114
Neutrophil %	76.38 ± 11.52	86.43 ± 7.94	-2.953	0.005*
HCT, %	39.45 ± 5.56	29.64 ± 6.62	5.159	< 0.001*
Platelet, x10^9/L	258 (226,348)	194.5 (91.25,258.75)	-2.881	0.004*
CRP, mg/L	39.74 (14.08,75.13)	183.49 (91.18,200)	-3.2	0.001*
PCT, ng/mL	0.06 (0.05,.54)	8.86 (1.29,24.37)	-4.185	< 0.001*
pH	7.41 (7.39,7.44)	7.41 (7.39,7.47)	-0.699	0.485
Na + , mmol/L	136(133.6,138.2)	140.5 (137.0,146.25)	-2.859	0.004*
K + , mmol/L	3.86 ± 0.49	3.69 ± 0.39	1.147	0.258
Glu, mmol/L	6.40 (5.01,8.73)	9.65 (7.38,12.00)	-2.648	0.008*
BUN, mmol/L	3.70 (3.19,6.13)	7.68 (5.32,15.46)	-3.053	0.002*
ANGPTL4, pg/ml	453.53 (362.27,830.89)	1441.70 (733.25,13,663.09)	-3.53	< 0.001*
CURB-65 score	1(0,1.5)	3(1,4)	-3.928	< 0.001*
PSI score	65(54,84.5)	123.5(95,140)	-4.231	< 0.001*

Quantitative data were presented as mean ± standard deviation or median (interquartile range). Count data were presented as number (%)

*nsCAP* Non-severe community-acquired pneumonia, *sCAP* Severe community-acquired pneumonia, *SBP* Systolic blood pressure, *DBP* Diastolic blood pressure, *MAP* Mean arterial pressure, *WBC* White blood cell, *HCT* Hematocrit, *CRP* C-reactive protein, *PCT* Procalcitonin, *Glu* Glucose, *Scr* Serum creatinine, *BUN* Blood urea nitrogen, *ANGPTL4* Angiopoietin-like 4, *PSI* Pneumonia severity index

* Indicates a significant value, *P* < 0.05

### Selection of the risk predictors

A total of 14 risk factors were noted to be statistically different between the two groups, which were further screened by ROC curve analysis. Among them, 4 risk factors with AUC > 0.8 were selected: CRP, PCT, ANGPTL4, and consciousness disorders (Table 2). As some medical records lacked detailed consciousness disorders grading scores and the scores were mainly dependent on subjective judgment in clinical practice, the variable of consciousness disorders was not included in the subsequent model development due to its error.

**Table 2:** ROC curve analysis of variables significantly different in univariable analysis

Variable	AUC	S.E	*P*-value	95% CI	
				Lower bound	Upper bound
CRP	0.800	0.072	0.001	0.658	0.941
PCT	0.886	0.048	< 0.001	0.792	0.980
Neutrophil %	0.757	0.079	0.006	0.602	0.912
Pulse	0.687	0.094	0.047	0.503	0.870
Platelet	0.229	0.076	0.004	0.080	0.378
Na +	0.768	0.084	0.004	0.605	0.932
Glu	0.749	0.078	0.008	0.597	0.901
BUN	0.787	0.083	0.002	0.625	0.949
SBP	0.286	0.082	0.023	0.125	0.446
DBP	0.098	0.048	< 0.001	0.004	0.191
ANGPTL4	0.832	0.059	< 0.001	0.715	0.948
HCT	0.135	0.068	< 0.001	0.001	0.269
Consciousness disorders	0.896	0.061	< 0.001	0.777	1.000
Pleural effusion	0.780	0.078	0.003	0.628	0.932

*ROC* Receiver operating characteristic, *AUC* Area under the curve, *S.E.* Standard error, *95%CI* 95% Confidence interval, *CRP* C-reactive protein, *PCT* Procalcitonin, *Glu* Glucose, *BUN* Blood urea nitrogen, *SBP* Systolic blood pressure, *DBP* Diastolic blood pressure, *HCT* Hematocrit

### Development and validation of a prediction model

**Fig. 1:**
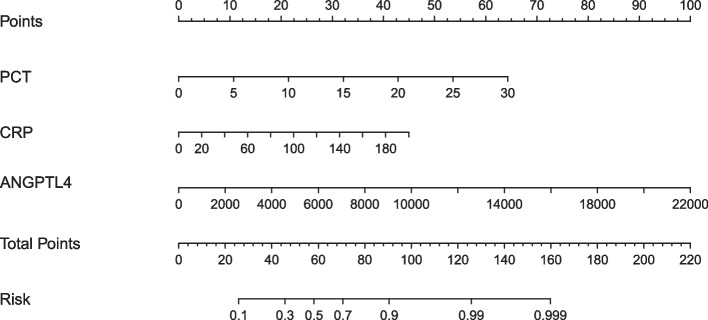
A nomogram developed for predicting the severity of community-acquired pneumonia. PCT, Procalcitonin; CRP, C-reactive protein; ANGPTL4, the serum expression of Angiopoietin-like 4

**Fig. 2:**
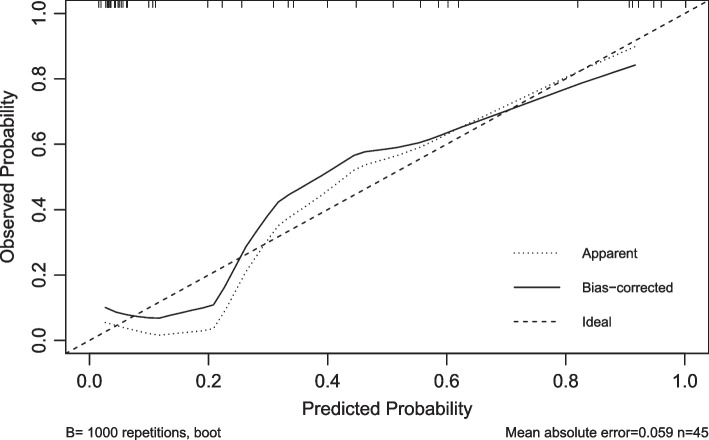
The calibration curve of the nomogram

**Fig. 3:**
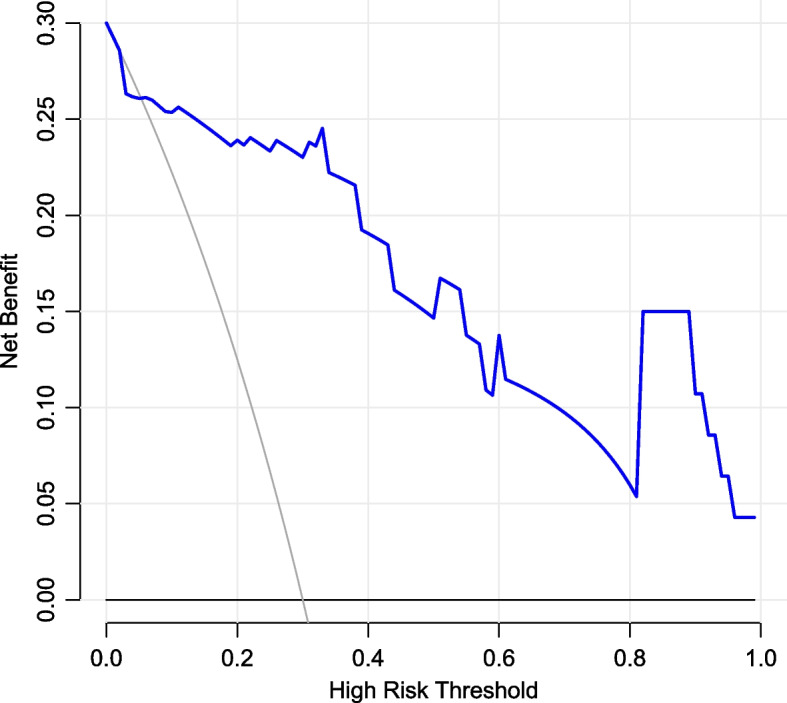
Decision curve analysis of the nomogram. The abscissa represents the threshold probability, and the ordinate represents the net benefit rate. The horizontal image indicates net benefit when all patients with CAP are not considered as developing into sCAP and not treated. The oblique image indicates net benefit when all CAP patients are considered as sCAP and treated. The blue line depicts the decision curve predicting sCAP probabilities

Finally, 3 risk factors (CRP, PCT, and ANGPTL4) were directly imported into the development of a multivariate logistic regression model, which is displayed as a nomogram in Fig. 1. The C-index by bootstrap resampling was 0.91, indicating a satisfactory internal validation. The Hosmer–Lemeshow test (χ2 = 10.13, *P* = 0.26) and the approximately fitting calibration curve suggested a satisfactory prediction accuracy (Fig. 2). The nomogram showed a great net benefit by DCA (Fig. 3), indicating its high clinical applicability.

### Comparison between the new prediction model with CURB-65 and PSI scores

**Fig. 4:**
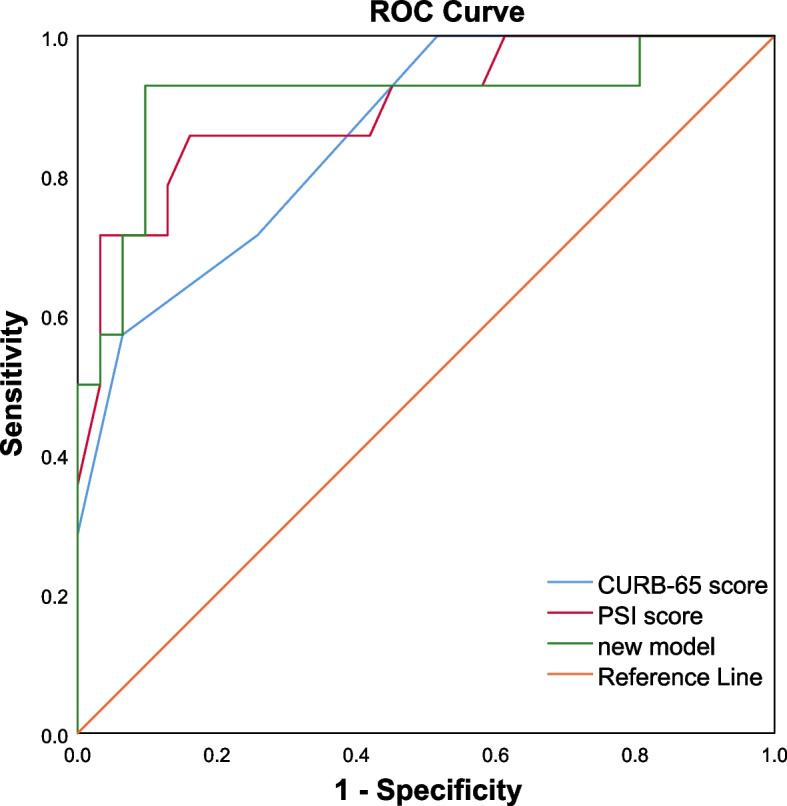
The ROC curves of the CURB-65 score, PSI score, and the new nomogram model. The new model, a multiple logistic regression model constructed by three factors: the serum expression of C-reactive protein, procalcitonin, and ANGPTL4, was visualized as nomogram in Fig. 1

As mentioned above, the new prediction model was developed by multivariate logistic regression analysis with 3 risk factors (CRP, PCT, and ANGPTL4), which was visualized as a nomogram to predict sCAP probability. As shown in Table 1, CURB-65 and PSI scores were statistically different between the two groups. The results of comparing the nomogram with CURB-65 and PSI scores for predicting the severity of CAP are presented in Table 3. The AUC values of CURB-65 score, PSI score, and the new prediction model were 0.857, 0.912, and 0.940, respectively. The AUC value of the new prediction model was greater than CURB-65 and PSI scores (Fig. 4), while the difference was not statistically significant (*P* > 0.05). By DeLong’s test, the *P*-value of AUC comparison between the new model with CURB-65 and PSI scores was 0.433 and 0.310. Both NRI and IDI comparing the new model with CURB-65 and PSI scores were higher than 0, whereas only NRI comparing the new model with CURB-65 score was statistically significant (NRI = 0.834, 95% confidence interval (CI): 0.252 ~ 1.416, *P* < 0.05). These results indicated that the accuracy of the new prediction model in evaluating the severity of CAP was higher than that of CURB-65 score.

**Table 3:** Comparison between the new prediction model and other scoring systems

Statistics	New model	CURB-65 score	PSI score
AUC (95%CI)	0.940(0.855,1)	0.857(0.745,0.969)	0.912(0.830,0.993)
*P*-value of AUC	Ref	0.433	0.310
NRI (95%CI)	Ref	0. 834(0.252, 1.416)	0.2256(-0.400, 0.852)
*P*-value of NRI	Ref	0.005	0.48
IDI (95%CI)	Ref	0.168(-0.040, 0.377)	0.0478(-0.140, 0.235)
*P*-value of IDI	Ref	0.114	0.618

New model, a multiple logistic regression model constructed by three factors: the serum expression of C-reactive protein, procalcitonin, and ANGPTL4, was visualized as nomogram in Fig. 1

*AUC* Area under the curve, *NRI* Net reclassification improvement, *IDI* Integrated discrimination improvement, *Ref* Reference group

## Discussion

Due to the rapid progression and high mortality of severe CAP, an accurate assessment of disease severity in the early-stage is critical in patient management, especially in guiding treatment strategies, such as ICU hospitalization, antimicrobial administration, and suitability for discharge [29]. In the present study, sCAP-associated risk factors were identified, of which 3 risk factors (ANGPTL4, CRP, and PCT) were selected, and a nomogram was developed, in order to predict the severity of CAP. The novel scoring system showed a greater prediction ability than CURB-65 score, and it is more appropriate for clinicians to accurately assess the severity of CAP, particularly in the early-stage.

ANGPTL4 is a secreted protein in host response to the hypoxic and nutrient-poor conditions, belonging to angiopoietin-like protein family. Native full-length ANGPTL4 (flANGPTL4) may release the N-terminal region (nANGPTL4) and the monomeric C-terminal portion (cANGPTL4) after proteolysis. As a multifunctional protein, ANGPTL4 is very active in angiogenesis and regulation of vascular permeability, energy homeostasis, wound healing, tumorigenesis, lipid and carbohydrate metabolism, and lung tissue integrity [26, 30–34]. The nANGPTL4 can serve as a lipoprotein lipase inhibitor to regulate lipid metabolism [35–37]. Moreover, carbohydrate metabolism is influenced by the regulation of the nANGPTL4 [38]. The cANGPTL4 induces endothelial cell disruption and compromises the vascular integrity by interacting with integrin α5β1, VE-cadherin, and claudin-5, which may further stimulate intracellular pathways that promote wound and tumorigenesis [39]. In recent years, several studies have confirmed the role of ANGPTL4 in lung tissue integrity [14, 27, 39–41].

Both in mice and patients with infectious pneumonia, Li et al. found that the spatiotemporal expression level of ANGPTL4, especially cANGPTL4 protein, was elevated in lung tissue injury accompanied by inflammation aggravation [27]. They also confirmed that influenza infection could upregulate ANGPTL4 expression level through a STAT3-mediated mechanism, which is consistent with Xu et al.’s findings [42], and STAT can be activated by interferon-gamma (IFN-γ) and interleukin 6 (IL-6) through viral infection triggering host responses. The above-mentioned results confirmed that ANGPLT4 can serve as a potential marker of lung diseases and ANGPLT4 expression level can be utilized to assess the severity of sCAP.

It was reported that ANGPTL4 upregulation was not limited to specific strain of influenza virus [27]. By comparing samples from coronavirus disease 2019 (COVID-19) patients and healthy individuals, Bhatraju PK et al. found that ANGPTL4 expression level in plasma was positively correlated with worse mortality, and ANGPTL4 was increasingly expressed in pulmonary epithelial cells and fibroblasts [41]. In addition to the infection by influenza and COVID-19 viruses, Li et al. demonstrated that immunoneutralization of cANGPTL4 could reduce pulmonary damage in secondary bacteria-infected mice [40]. It indicates that ANGPTL4 has the potential to serve as a CAP marker and host-directed targeted therapy whether for viral infection, bacterial infection or other pathogens. In the present study, the pathogens in the lower respiratory tract of all patients were considered to be bacteria, because all patients were clinically improved after receiving antibacterial drugs. However, the role of viral pathogens in CAP has noticeably attracted clinicians’ attention [5]. Regarding the rapid variation of pathogens and the difficulty of successful etiological culture, the relative stability of host response to infection is increasingly recognized as a target for diagnosis and treatment development [16–18, 43–45]. The serious consequence of infection is lung injury, including inflammatory infiltration, vascular leakage, the damage of structural integrity and pulmonary edema. Therefore, ANGPTL4 can serve as a potential biomarker to assess the severity of CAP based on host response after infection.

Moreover, several studies have shown that immunoneutralization of ANGPTL4 could significantly reduce vascular leakage and tissue integrity disruption and promote lung recovery [14, 27, 40, 41], which not only emphasizes the potential of ANGPTL4 as a marker for assessment of pneumonia severity, but also induces a promising therapy, targeting ANGPTL4 for lung recovery.

There is a divergence in finding concerning ANGPLT4 expression level in non-infectious lung diseases. In contrast to Li et al.’s results that cANGPTL4 expression level could not be detected by immunoblot analysis in the lung sample with obstructive pneumonia [27], it was reported that ANGPTL4 expression level in serum of patients with chronic obstructive pulmonary disease (COPD) was upregulated [46, 47]. Liu H et al. used cigarette smoke extract to stimulate lung dysfunction and found that ANGPTL4 knockdown could relieve oxidative stress, apoptosis, and smoking-induced lung dysfunction by inhibiting nicotinamide adenine dinucleotide phosphate (NADPH) oxidase 2 (NOX2) and blocking JNK/p38 MAPK signaling pathway, which is consistent with other research results that ANGPTL4 could play a key role in pulmonary function and systematic inflammation [48]. Thus, ANGPTL4 expression level could also increase in non-infectious pneumonia cases. Distinguishing infectious and non-infectious pneumonia by ANGPTL4 expression level alone still seems to be a diagnostic challenge, highly influencing therapeutic strategies. Thus, developing a prediction model combining serum ANGPTL4 level and other clinical diagnostic indices is advantageous to accurately diagnosis and treat CAP.

### Study limitation

In the present study, a nomogram was developed using serum levels of three sCAP-associated risk factors (ANGPTL4, CRP, and PCT). Some studies demonstrated that ANGPTL4 expression level also increased in patients with cancer, vascular pathologies, cardiovascular disease, and COPD [39, 46, 47, 49]. In the present study, patients with such diseases were not excluded, indicating that the detection of ANGPTL4 expression level might not be fully associated with CAP. Besides, it was reported in the previous study that ANGPTL4 can regulate the activity of lipoprotein lipase activity and hypertriglyceridemia [50]. But whether the level of hypertriglyceridemia and the use of fibrates influence the diagnostic value of ANGPTL4 have not been identified in present study. Therefore, in the future study, it is necessary to set subgroups based on comorbidities and the use of drugs to exclude the influence of confounding factors on the research results. Notably, it was reported that the expression level of cANGPTL4, one of isoforms of ANGPTL4 proteolysis, was mainly upregulated in patients with pneumonia [27]. Previous studies have shown that different pathogens with different ANGPTL4 protein expression profiles and different ANGPTL4 isoforms are associated with different functions [26, 27]. The ELISA method was used to measure the ANGPTL4 expression level in the present study, which has been confirmed to have high affinity for cANGPTL4 with less affinity for full-length and minimal affinity for nANGPTL4 [41]. Although ELISA is still the first option for detection of ANGPTL4 expression level, it cannot completely separate different ANGPTL4 isoforms [30]. Future studies are suggested to concentrate on the methods of detection, isolation, and identification of different ANGPTL4 isoforms, so as to improve the accuracy of experimental results.

Cross-sectional studies are often used to characterize diseases or other phenomena, to explore the underlying patterns of occurrence, and to provide clues for further follow-up researches. The aim of this study was to clarify whether ANGPTL4 was helpful in diagnosing sCAP. However, our cross-sectional study lacked dynamic changes in ANGPTL4 and cannot yet causally correlate it with sCAP. Nonetheless, on the basis of previous researches [14, 26, 27, 39–41], our study showed that ANGPTL4 was also able to identify sCAP well by constructing a multivariate logistic regression analysis, breaking the ice for subsequent studies.

As a tool to transform a statistical model into a visual graph, nomogram is simple and intuitive enough for clinicians to accurately evaluate the severity of CAP [28, 51, 52]. And thankfully the prediction performance of our nomogram was satisfactory. But it is undeniable that the sample size of our study was indeed limited. Though the small sample size may make the regression model unstable, we still establish this nomogram for the reason that the goodness of fit was acceptable by the Hosmer–Lemeshow test (χ2 = 10.13, *P* = 0.26). Besides, before performing multiple logistic regression analysis, we have carefully filtered the disruptive variables by difference analysis and professional evaluation. And the nomogram showed good prediction ability after internally verified by bootstrap resampling. In this study, we only employed a few patients to conduct primary research on the influence of ANGPTL4 to sCAP assessment. Therefore, before applying this diagnostic model to clinical practice, external verification is essential, which requires to expand the sample size in the future study.

Besides, when using ROC analysis to screen risk factors, the variable of consciousness disorders was excluded due to its large subjective errors in data collection, which might lead to the omission of some important risk factors. When compared with CURB-65 and PSI scores in terms of prediction performance, the new prediction model had a higher AUC, while there was no statistically significant difference. The AUC was utilized to evaluate the discriminatory potential of the scoring system. ROC analysis, belonging to qualitative analysis, is not sensitive enough to the change of the estimated risk. However, as quantitative analysis methods, NRI and IDI were introduced to further compare the prediction performance between these scoring systems [53]. Both NRI and IDI comparing the new model with CURB-65 and PSI scores were higher than 0, while only NRI comparing the new model with CURB-65 score was significantly higher than 0. The small sample size could be one of the reasons for this result. This novel scoring system should be further validated in multicenter prospective studies. The results of the present study revealed that the new prediction model based on the serum expression of ANGPTL4 has a certain improvement effect compared with CURB-65 score.

## Conclusions

The ANGPTL4 has a great potential to be a diagnostic biomarker for CAP. A new prediction model for the severity of CAP was built based on ANGPTL4 expression level. Large-scale multicenter studies are still needed to verify these results before clinical application.

## Data Availability

The data underlying this article may be available from the corresponding author on reasonable request.
